# Supplementary material for: Substantial Effect of Water on Radical Melt Crosslinking and Rheological Properties of Poly(ε-Caprolactone)
Source: Polymers (Basel). 2021 Feb 4;13(4):491. doi: 10.3390/polym13040491 (PMC7915490; doi:10.3390/polym13040491)
Supplement: Supplementary file 1 [file polymers-13-00491-s001.pdf]

polymers

Supporting information

Substantial effect of water on radical melt crosslinking and rheological properties of poly(ε-caprolactone)

Angelica Avella [1], Rosica Mincheva [2], Jean-Marie Raquez [2] and Giada Lo Re [1,*]

[1] Department of Industrial and Materials Science, Division of Engineering Materials, Chalmers University of Technology, SE-412 96 Gothenburg, Sweden; avella@chalmers.se (A.A.); giadal@chalmers.se (G.L.R.)
[2] Laboratory of Polymeric and Composite Materials (LPCM), Center of Innovation and Research in Materials and Polymers (CIRMAP), University of Mons (UMONS), B-7000 Mons, Belgium; rosica.mincheva@umons.ac.be (R.M.); jean-marie.raquez@umons.ac.be (J-M.R.)
* Correspondence: giadal@chalmers.se (G.L.R.)

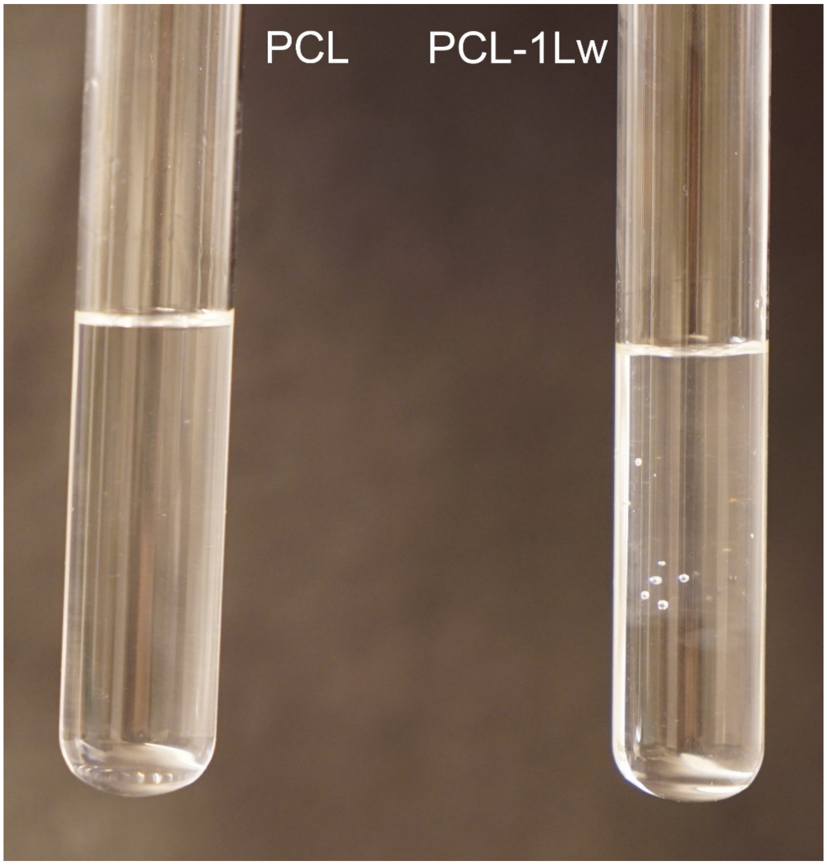

Figure S1. Photographs of test tubes containing neat poly(ε-caprolactone) (PCL) and PCL reacted with 1 wt.% peroxide in the presence of water (PCL-1Lw), after 24 h in solution with dichloromethane. PCL-1Lw shows a large gel fraction, disclosed by the opalescence of the floating supernatant, which appears more viscous and with visible entrapped air bubbles (right photograph), in contrast with the transparent and neat PCL, completely soluble in dichloromethane (left photograph).

Table S1. Thermal properties of neat and reacted PCL detected by Thermogravimetric analysis (TGA) and Differential Scanning Calorimetry (DSC). TGA: Onset temperatures of degradation ($T_{5\%}$) evaluated at 5 % weight loss; degradation temperature (T_d) evaluated as the peak temperature of DTG; char residue at 550 °C. DSC: Glass (T_g) and melting (T_m) temperatures detected from the second heating scan; melting enthalpy (ΔH_M) and crystallinity (χ_{DSC}) detected from the melting peak in the second heating scan (from 15 to 65 °C); crystallization temperature (T_c) detected from the cooling scans.

Material	$T_{5\%}$ [°C]	T_d [°C]	Char [%]	T_g [°C]	T_m [°C]	ΔH_M [J·g^{-1}]	χ_{DSC} [%]	T_c [°C]
PCL	363	400	1	-62	57	63.73	47.0	35
PCL-0.1L	290	398	1	-63	57	62.51	46.1	30
PCL-0.25L	304	398	3	-65	57	60.36	44.5	31
PCL-0.5L	331	398	2	-62	57	62.9	46.4	37
PCL-0.5Lw	325	398	2	-61	57	59.23	43.7	36
PCL-1L	345	398	0	-62	57	63.26	46.6	35-40
PCL-1Lw	347	399	2	-58	59	61.34	45.2	41

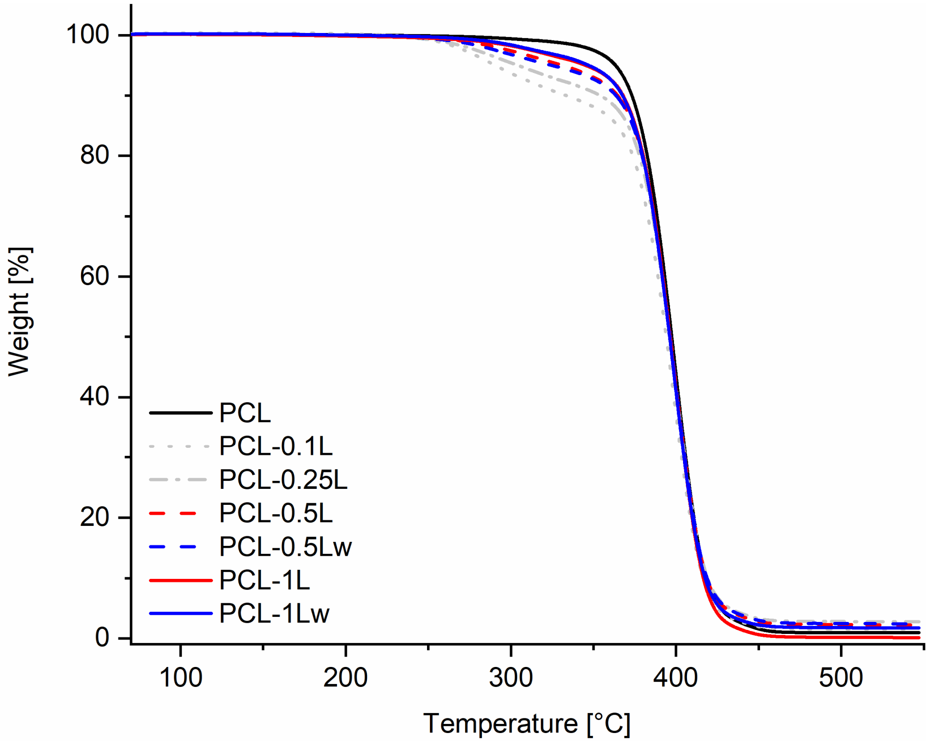

Figure S2. Curves from thermogravimetric analysis (TGA) of neat and reacted PCL with different wt.% of peroxide during dry (PCL-xL) or water-assisted (PCL-xLw) melt processing.

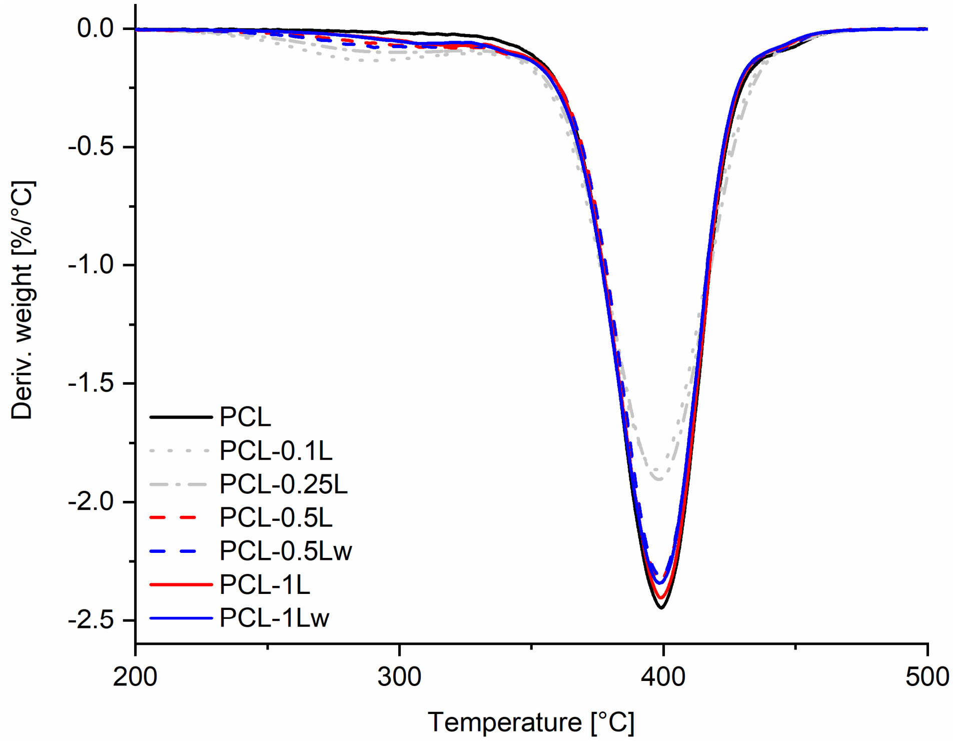

Figure S3. Derivative thermogravimetric (DTG) curves of neat and reacted PCL.

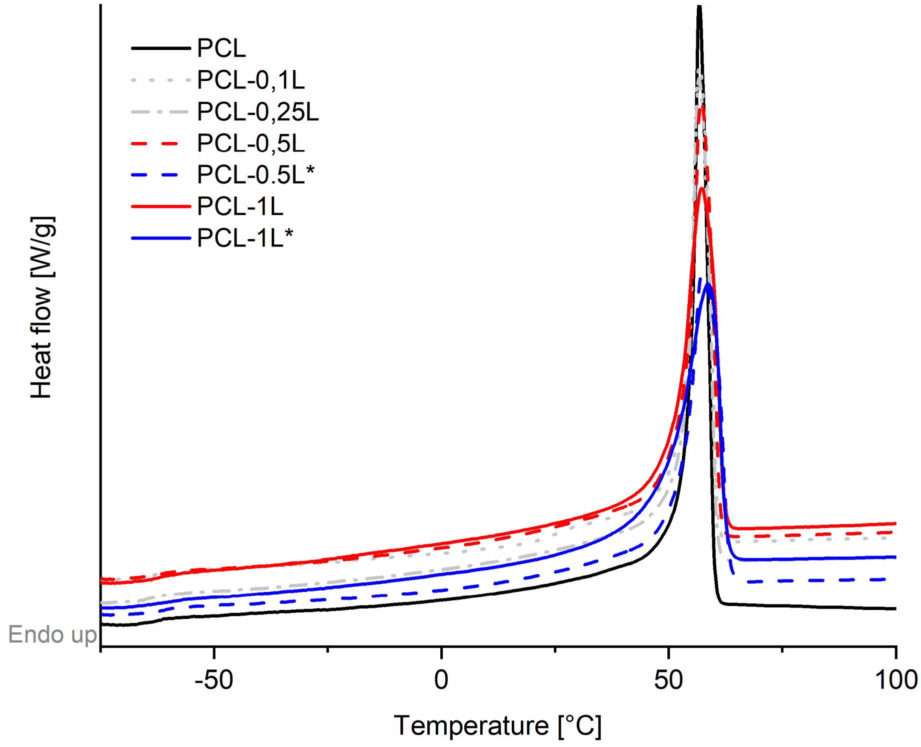

Figure S4. Thermograms from second heating scan of Differential Scanning Calorimetry (DSC) of neat and reacted PCL.

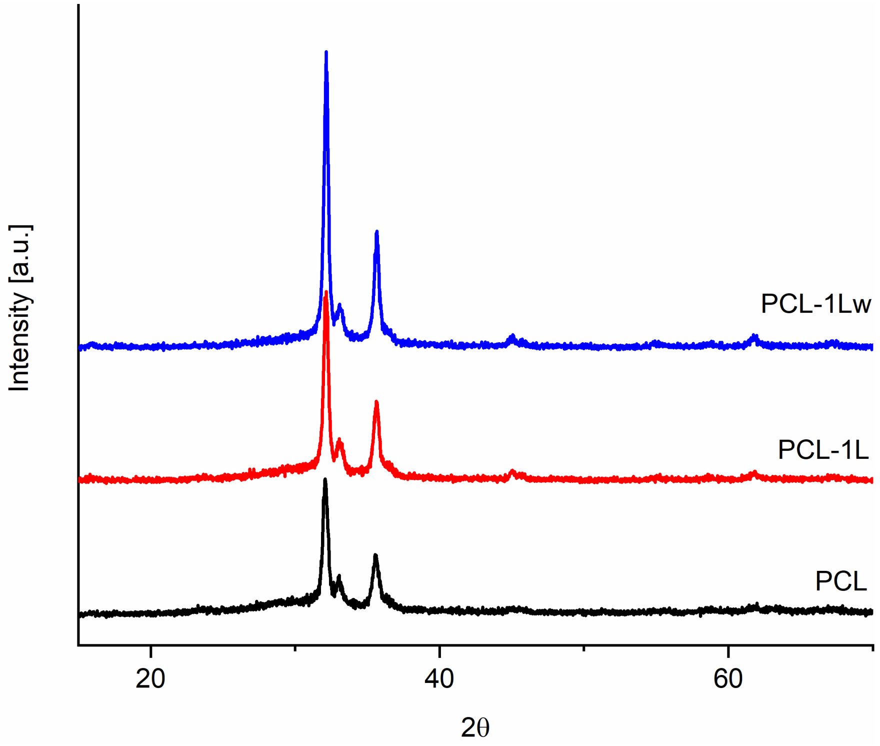

Figure S5. X-Ray diffractograms of PCL and PCL reacted with 1 wt.% peroxide during dry (PCL-1L) or water-assisted (PCL-1Lw) melt processing.

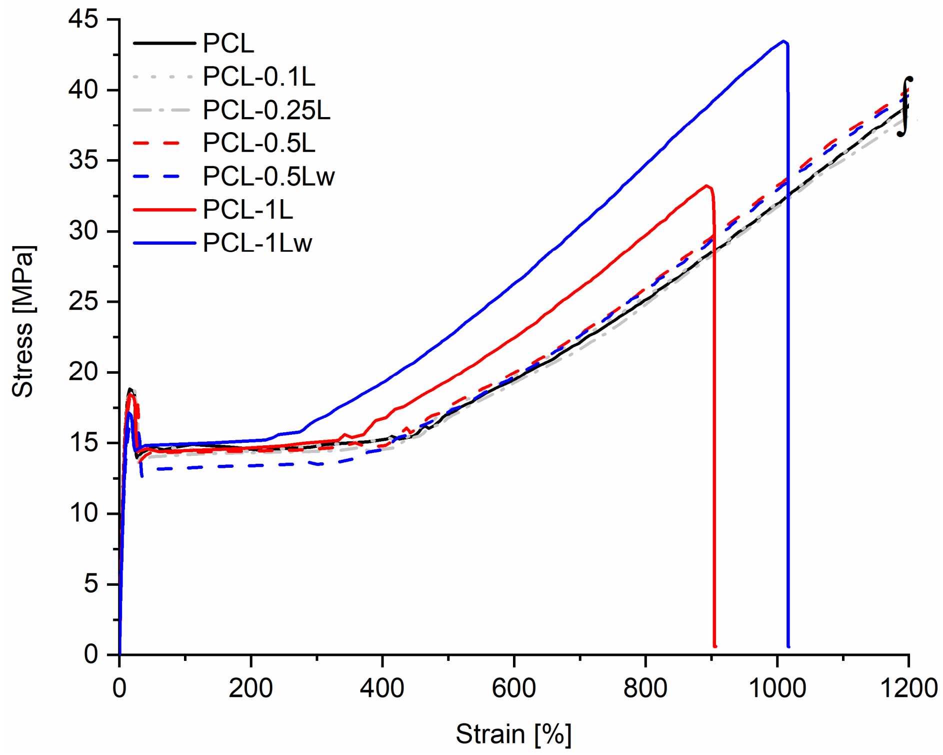

Figure S6. Representative stress-strain curves from tensile tests at room temperature neat and reacted PCL.

Table S2. Mechanical properties assessed from tensile tests at room temperature. Each value represents the average of 5 measurements with the standard deviation. *Data extracted at the upper limit of the instrument.

Material	Young's modulus [MPa]	Yield stress [MPa]	Ultimate tensile strength [MPa]	Elongation at break [%]
PCL	284 ± 5	19.2 ± 0.5	38.7 ± 0.6 *	1200 *
PCL-0.1L	262 ± 3	18.7 ± 0.4	39 ± 0.5*	1200*
PCL-0.25L	262 ± 2	18.5 ± 0.1	39.1 ± 0.9*	1200*
PCL-0.5L	248 ± 6	18.4 ± 0.1	38.8 ± 2.4 *	1200*
PCL-0.5Lw	218 ± 8	16 ± 0.4	39.8 ± 0.1 *	1200*
PCL-1L	242 ± 13	17.3 ± 1	33.6 ± 1.3	941 ± 57
PCL-1Lw	225 ± 14	17.1 ± 0.5	44 ± 1.2	1035 ± 48
